# Policies and clinical practices relating to the management of gestational diabetes mellitus in the public health sector, South Africa – a qualitative study

**DOI:** 10.1186/s12913-018-3175-x

**Published:** 2018-05-10

**Authors:** Lorrein Shamiso Muhwava, Katherine Murphy, Christina Zarowsky, Naomi Levitt

**Affiliations:** 1 Department of Medicine, University of Cape Town, Cape Town, South Africa; 2 Chronic Diseases Initiative for Africa (CDIA), Cape Town, South Africa; 3 University of Montreal, Hospital Research Centre and University of Montreal School of Public Health, Montreal, Canada; 4 School of Public Health, University of the Western Cape, Cape Town, South Africa

**Keywords:** Gestational diabetes, Type 2 diabetes, Health services, Health system, Policy, South Africa

## Abstract

**Background:**

Women with a prior gestational diabetes have an increased lifetime risk of developing type 2 diabetes. Although post-partum follow-up for GDM women is essential to prevent progression to type 2 diabetes, it is poorly attended. The need for health systems interventions to support postpartum follow-up for GDM women is evident, but there is little knowledge of actual current practice. The aim of this study was to explore current policies and clinical practices relating to antenatal and post-natal care for women with GDM in South Africa, as well as health sector stakeholders’ perspectives on the barriers to -- and opportunities for -- delivering an integrated mother - baby health service that extends beyond the first week post-partum, to the infant’s first year of life.

**Methods:**

Following a document review of policy and clinical practice guidelines, in-depth interviews were conducted with 11 key informants who were key policy makers, health service managers and clinicians working in the public health services in South Africa’s two major cities (Johannesburg and Cape Town). Data were analysed using qualitative content analysis procedures.

**Results:**

The document review and interviews established that it is policy that health services adhere to international guidelines for GDM diagnosis and management, in addition to locally developed guidelines and protocols for clinical practice. All key informants confirmed that lack of postpartum follow-up for GDM women is a significant problem. Health systems barriers include fragmentation of care and the absence of standardised postnatal care for post-GDM women. Key informants also raised patient - related challenges including lack of perceived future risk of developing type 2 diabetes and non-attendance for postpartum follow up, as barriers to postnatal care for GDM women. All participants supported integrated primary health services but cautioned against overloading health workers.

**Conclusion:**

Although there is alignment between international guidelines, local policy and reported clinical practice in the management of GDM, there is a gap in continuation of care in the postpartum period. Health systems interventions that support and facilitate active follow-up for women with prior GDM are needed if high rates of progression to type 2 diabetes are to be avoided.

**Electronic supplementary material:**

The online version of this article (10.1186/s12913-018-3175-x) contains supplementary material, which is available to authorized users.

## Background

Gestational diabetes mellitus (GDM), that is diabetes diagnosed in the second or third trimester of pregnancy, affects up to 28% of pregnancies globally [1, 2]. Women with GDM have a significantly increased lifetime risk (≥ 70%) for developing type 2 diabetes, a 3-fold risk of developing the metabolic syndrome and an increased long-term risk of developing cardio-vascular disease (CVD) [3, 4]. In addition, children born of women with GDM are susceptible to impaired glucose tolerance and obesity in adulthood [5–8]. International recommendations for the management of GDM emphasise the need for post-partum follow up and care, including an Oral Glucose Tolerance Test (OGTT) from 6 to 12 weeks postpartum and continued support for lifestyle change [9]. However, compliance with these recommendations is reported to be low [10]. In South Africa, as is the case elsewhere, most women with GDM are lost to follow-up after delivery [11, 12].

The sparse data available from 11 of the 55 countries in Sub-Saharan Africa (SSA) reveal a wide range in the prevalence of GDM, from 0 to 14% [13, 14]. While South Africa’s exact prevalence is unknown, it is currently estimated to be greater than 15% [13]. A recent SA study reported a GDM prevalence of 25.8% with universal screening and 15.2% with selective risk factor screening using the International Association of Diabetes in Pregnancy Study Groups (IADPSG) criteria [15]. The lack of uniformity in GDM screening and diagnosis practices in SA is concerning. Anecdotal reports indicate that GDM screening is not consistent and many women remain undiagnosed. However, the feasibility of universal screening for GDM in SA, given resource constraints, is yet to be established. In addition, the reported high rates of GDM are likely to be driven by the fact that the country has the highest rate of obesity and overweight in sub-Saharan Africa: up to 70% of women are estimated to be overweight or obese and the prevalence of obesity among women has risen from 30% in 1998 (SADHS) to 42% in 2013 [16, 17]. Given the expectations of increasing rates of GDM in SA and the high risk of progression to type 2 diabetes among this group, there is an urgent need to develop interventions with GDM women to prevent or delay progression to type 2 diabetes.

The South African public health system provides healthcare services to approximately 84% of the population and is overwhelmed by the multiple disease burden including chronic infectious diseases and NCDs [18]. A minority of the population has access to and can afford private healthcare services, which are typically far better resourced. The proposed IINDIAGO project – an acronym for “An integrated health system intervention aimed at reducing type 2 diabetes in disadvantaged women after gestational diabetes in South Africa”, is therefore situated in the context of the public health sector and focuses on women from disadvantaged communities, who would benefit from interventions to improve health services.

Several studies and systematic reviews show that lifestyle interventions for women with prior GDM, through diet and exercise are effective in reducing their risk of developing type 2 diabetes, improving health outcomes for both the mother and baby [8, 19, 20]. In addition, lifestyle interventions for high-risk groups for type 2 diabetes (including people with obesity, impaired glucose tolerance, and impaired fasting glucose and GDM) have been found to be cost-effective [21]. Where interventions have had little success, this has been attributed to inadequate post-partum follow up and support and a poor understanding of context in the development of interventions. However, many of these intervention studies have been conducted in developed countries and have not yet been demonstrated to be feasible or effective in a resource limited setting [8, 19, 20].

Formative research is increasingly recognised and undertaken as an essential process in the development of health behaviour change interventions [22–26]. The formative research process is a critical component to intervention design which allows for context-driven information gathering that will subsequently guide and inform the development of an intervention that best fits the targeted beneficiaries [22]. This paper reports on one component of the formative research for the IINDIAGO project, which aims to develop and evaluate a novel health system intervention to reduce the risk of subsequent type 2 diabetes among women with recent GDM, that can be integrated into existing health services in South Africa. The formative research for the IINDIAGO project will assist the project team to understand the context in which the proposed intervention is to be initiated, including cultural, social, health system and contextual factors, which influence health seeking and lifestyle related behaviour [23, 27].

As little was currently known about the policies and clinical practices relating to the management and care for women with GDM in South Africa, the purposes of this study were two-fold. The first objective was to explore the existing policies and reported clinical practices relating to antenatal and post-natal care for women with GDM in the public health sector in South Africa. The second objective was to identify the barriers to -- and opportunities for -- delivering the intervention - an integrated GDM mother - baby health service that extends beyond the first week post-partum, through the infant’s first year of life.

The results from this study will contribute to the design and implementation of a feasible and sustainable intervention for women with GDM postpartum, within the context of existing public health services in South Africa.

## Methods

### Study design

We conducted a qualitative study consisting of; (i) a document review of policy documents and clinical practice guidelines for the screening, diagnosis and management of GDM and (ii) semi-structured in-depth interviews with key informants on the management and care for women with GDM during pregnancy and postpartum within the context of public sector health services in South Africa. The use of qualitative methods allowed for an in-depth assessment of the barriers and facilitators for implementation of the proposed intervention from the perspective of those with responsibility for GDM care and policy.

### Study setting

The study was conducted in the context of three urban public sector hospitals in Cape Town (Western Cape province) and Soweto (Gauteng province), South Africa, which are classified as secondary (Level 2) and tertiary (Level 3). The secondary hospital is a regional hospital, which provides health services to women with complicated pregnancies referred from Midwife Obstetric Units (MOUs) around Cape Town. The two tertiary hospitals have dedicated antenatal diabetes clinics and provide health services to patients referred from primary and secondary health facilities mainly within the Western Cape and Gauteng provinces but not limited to provincial boundaries. Due to the recognized high standards of care at these hospitals, it is common for pregnant women from rural areas to migrate to these provinces for the duration of their pregnancy, to access antenatal care services.

### Study sample

Criterion-based and sequential referral sampling were used to identify key informants who had expert knowledge on clinical practice guidelines and policies on GDM and/or had experience in providing clinical care for GDM women in the three public sector hospitals in Cape Town and Soweto, South Africa. Criterion sampling identifies individuals with specific characteristics of interest that would enable the researcher to obtain in-depth information to answer the research question [28, 29]. Informants were therefore selected based on their positioning in the field and/or their potential for influencing policy. Key informants comprised policy makers, health service managers from the Department of Health and clinicians working in the public health services. Sequential referral sampling was used to follow up on other important key informants recommended by respondents. Participants were recruited until saturation was reached. Invitations to participate in the study were sent via email and followed up telephonically.

### Data collection

Data collection was conducted in two phases. Firstly, a document review of international and local guidelines pertaining to the management of GDM was conducted. Documents for review consisted of various policy documents, clinical practice guidelines for the screening, diagnosis and management of GDM in SA and educational materials provided to women with GDM. The key informants provided most of the documents during the interviews and others were sourced from the websites of the national and provincial Departments of Health. The documentary reviews provided the background needed to contextualise the data from the key informant interviews.

Following the document review, interviews were conducted by a trained qualitative researcher, in a private location most convenient to each respondent. These were typically in their offices at the hospital, clinic or health departments. Prior to the interview, each key informant was given the draft project proposal and asked to subject it to critical review. A discussion guide (Additional file 1) was used, but each respondent was also given opportunities to raise other issues that may not have been anticipated by the researcher. The discussion guide was slightly modified as the study progressed on the basis of new issues arising in previous interviews. The discussion guide included questions on the following topics: current policy and clinical practice guidelines for the management of GDM, implementation and oversight of the policy guidelines, postpartum care for women after GDM, views on the proposed intervention and potential barriers to and opportunities for delivering the proposed integrated mother - baby intervention in the Well Baby Clinic. Each interview lasted between 45 min and 1 h. All interviews were audio recorded and transcribed verbatim. All audio recordings were stored in a locked cabinet with restricted access.

### Data analysis

Content analysis was used to identify and summarise essential information from the sourced documents relating to the management of GDM and postnatal follow-up care for women after GDM. The documentary reviews provided triangulation of much of the information emerging from the key informant interviews.

**Fig. 1:**
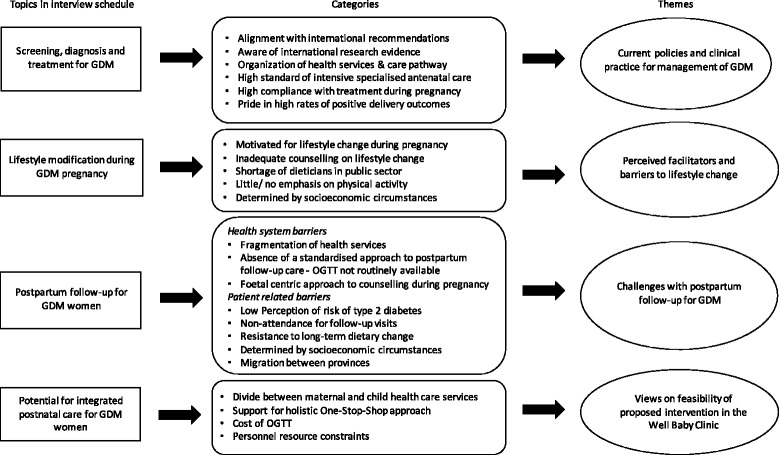
The coding process illustrating focus areas, categories and key thematic areas. A summary of the overall coding process moving from the focus areas in the discussion guide, to categories and key thematic areas

Data from key informant interviews were also analysed using qualitative content analysis [30, 31]. Firstly, all transcripts were read to get a general overview of the data. They were then re-read closely to identify codes, categories and themes. A qualitative data analysis software package, NVivo 11, assisted in managing and organising the data. The data was scrutinised for both deductive and inductive codes/ categories (i.e. predetermined issues relating to the research questions, as well as unanticipated, emergent issues). Once a certain number of transcripts had been analysed, a coding framework was then developed and applied across the rest of the data set, with various revisions. Two researchers were involved in analysing the data and reviewing final categories. The overall coding process moving from the focus areas to categories and key thematic areas is illustrated in Fig. 1.

Written informed consent was obtained from each participant prior to the interviews and filed for safekeeping. Each participant was fully informed as to the purpose and procedures of the research study and assured that their names would not be used in the write up of research findings. The need for a digital voice recorder was explained and permission was obtained before it was used.

## Results

Between July and November 2015, in-depth interviews were conducted with a total of 11 key informants: 2 key policy makers, including 1 health service manager from the Department of Health; 2 public health specialists and 7 clinicians working in the public health services. All those contacted consented to participation in the study. The results from the interviews are described under four main headings, which follow the key areas of investigation outlined in the discussion guide.

### I. Current policies and clinical practice for the management of GDM

The review of documents found that the three hospital sites follow the WHO criteria [32], as well as the UK National Institute for Health and Care Excellence (NICE) [33] guidelines for GDM diagnosis and management during pregnancy. In addition, there are national and provincial guidelines for screening, diagnosis and treatment for GDM i.e.; the National Guidelines for Maternity Care in South Africa [34] and the provincial Western Cape ‘Diabetes in Pregnancy’ guidelines [35]. The National Department of Health has adopted selective risk-factor screening based on the presence of risk factors which include obesity (BMI > 30 kg/m^2^), repeated glycosuria, previous GDM, age > 40 years and family history of diabetes (first-degree relative) [36]. Consistent with the international WHO guidelines [37], women with any of these risk factors should be offered an OGTT and GDM is then diagnosed on the basis of a fasting plasma glucose level of 5.6 mmol/litre or a 2-h plasma glucose level of 7.8 mmol/litre or above. Antenatal clinics at the primary care level also have specific protocols developed from the overarching national policy guidelines, which are used in clinical practice. The guidelines in South Africa have been developed through consultation with clinicians and other experts and are based on international research evidence. Participants highlighted the importance of research evidence in policy development and clinical practice and made reference to landmark empirical studies [38, 39] that have influenced practice.

#### Organisation of health services and the care pathway

**Fig. 2:**
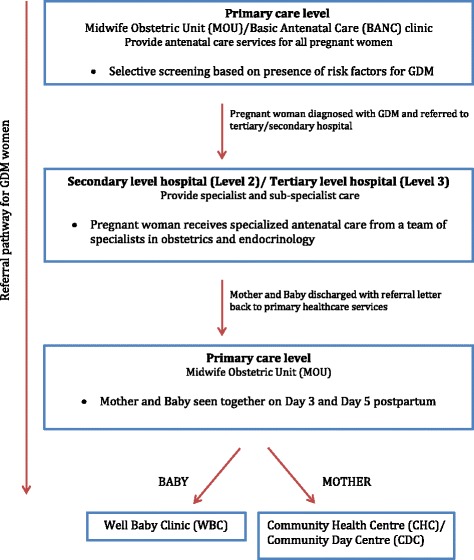
Levels of care and referral pathway for GDM women in South Africa. An overview of the organisation of health services and the referral pathway for GDM women in the public health services

**Fig. 3:**
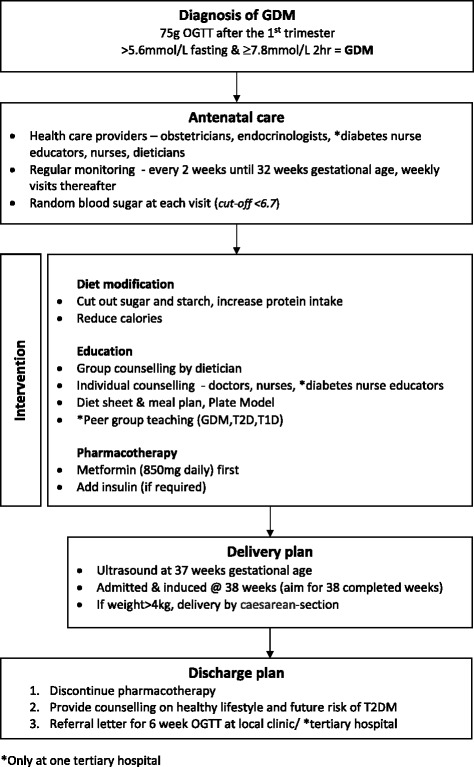
Management and care plan for women with GDM at 2 tertiary and 1 secondary hospital in South Africa. A summary of the management of GDM in the study settings, which entails one or a combination of (i) counselling on diet and physical activity by the doctors, nurses (and a dietician where available), (ii) oral agents such as metformin and (iii) insulin where necessary

The organisation of health services and the referral pathway for GDM women in the public health services in SA is summarised in Fig. 2. According to the document review and interviews; pregnant women first present for antenatal care either at a local MOU or Basic Antenatal Care (BANC) clinics, which are birthing units based in the community and run by midwives (Fig. 2). The MOU and BANC clinics provide similar primary health care services under provincial or local municipal governance respectively. Cape Town, like several other urban settings in South Africa has dual authority over health services – provincial Department of Health and the local municipal government – each providing somewhat independent governance over a number of community-based health services with their own set of guidelines. In both the MOUs and BANC clinics, the pregnant woman is screened for GDM on the basis of the presence of risk factors described above. Upon diagnosis, the pregnant GDM woman is referred for her remaining antenatal care and delivery at the nearest secondary or tertiary level hospital. At the tertiary level, women receive intensive specialist care and medical management from a team of obstetricians, endocrinologists, dieticians and nurses (Fig. 3). The Soweto study site also has a team of diabetes nurse educators who provide counselling and health education. In the Cape Town setting, women with Impaired Glucose Tolerance (IGT), receive antenatal care, including delivery at a secondary level hospital. The primary form of intervention for women with GDM is lifestyle modification. According to the key informants, antenatal care for women with GDM is of high standard with positive foetal and maternal outcomes. At discharge, women are counselled by their health care provider about their risk of developing type 2 diabetes in future and are advised to attend a follow-up visit at their nearest clinic or community health centre (CHC), within 6 to 12 weeks for an OGTT to determine whether they have type 2 diabetes postpartum. However, there are inconsistencies in the referral process from tertiary to primary care level and the OGTT is seldom offered at primary care level to women with prior GDM.

Although the management and care for women with GDM is in line with international guidelines, continuation of care in the postpartum period is problematic. In the first 10 days postpartum, the mother and baby typically receive care (e.g.; wound care after delivery through Caesarean -section and breastfeeding advice) at the MOU or BANC clinic, where she originally registered for antenatal care. Thereafter, the baby receives care at the Well Baby Clinic, which provides baby-feeding counselling, development assessment, weight monitoring and immunization, whilst the GDM mother is expected to receive any further care at her nearest primary health care clinic. Some clinicians felt their hospitals did not have the capacity to offer GDM women an OGTT postpartum; hence they follow policy recommendations by referring women to primary level clinics within their local communities for postpartum follow-up care.

The following section provides a summary of the results of the key informant interviews as depicted in Tables 1 and 2 below.

**Table 1:** Results of key informant interviews by categories and illustrative quotes

Key findings	Quotes from key informants
Theme 1: Perceived facilitators and barriers to lifestyle change	
Facilitators of lifestyle modification	
(i) Concern for the health of the unborn baby	*“The patients are well motivated in pregnancy, they do change because they want a live baby” - Diabetes nurse educator*
	*“Their compliance for the very reason that they want a healthy baby is much higher. It doesn’t mean that it stays like that postpartum” – Professor, Obstetrician 1*
	*“Pregnancy is a really good time to intervene because they are so worried about the baby and sometimes a bit shocked that something they can do or not do can harm their baby” – Professor, Obstetrician 2*
Barriers to lifestyle modification	
(i) Inadequate dietary counselling due to shortage of dieticians in the public sector	*“Ideally they should see a Dietician, but not everybody has access to a Dietician, and there are too many patients” – Obstetrician 3* *“Actually the people that should control nutrition are nurses because there are so few dieticians. They (dieticians) are either at a hospital or at a sub-district level but day to day, it’s the nurses who are encountering patients” –Professor, Public health specialist 1* *“Dieticians are a rare and scarce resource in this hospital and most of them do not have time to go into pregnancy work” - Professor, Obstetrician 4* *“If the dietician is not available, we take over, but the Dietician comes to do her part as well. The Dieticians come and go, they train, qualify, work for a bit, and then they usually go into private practice, I guess.” – Professor, Obstetrician 1* *“In discussions with the Department of Health around this, I think there is a sense that it was slightly ambitious to expect the nursing staff to do a full, kind of, dietary counselling intervention, and that actually the Dietician needs to be doing that...” – General practitioner, Medical Anthropology Researcher* *“It’s group counselling because we are limited, which is unfortunate because,” Obstetrician 5* *“We give them general advice but of course that probably isn’t enough” –Professor, Obstetrician 1*
(ii) Lack of full understanding of healthy diet requirements	*“A lot of the patients I don’t think actually understand what needs to be done in terms of the diet to be able to deal with this” – Obstetrician 5*
(iii) Lack of interventions for physical activity	*“I don’t think there is enough spoken about exercise actually, I think it could be useful” – Professor, Obstetrician 2*
(iv) Affordability of healthy food	*“We’ve got two groups of women, one who has resources and can try to follow the advice, most of those women do, they’ve really taken up the idea that vegetables are important, you shouldn’t drink alcohol and you should avoid sugar but the rest of the women, cost is the main factor in what they can do” – General practitioner, Medical Anthropology Researcher*
	*“I think also it’s just the reality of going home to limited financial resources” – Obstetrician 5*
	*“It’s really expensive to eat healthy; you can’t do it, because all the cheap food you can afford is junk food” – Professor, Public health specialist 1* *“I am just quite pessimistic about how much people can acquire a healthy diet, if they are below a certain income” –Professor, Public health specialist 2*
Theme 2: Challenges with postpartum follow-up for GDM women	
Health system barriers	
(i) Absence of a standardized postnatal care approach for GDM women	*“You are probably going to find that the policy (management of diabetes during pregnancy and postpartum) is not being implemented at primary care level. Part of the problem is that the diabetes policy is aimed at the doctors at the hospitals and the health care providers at the primary care clinic don’t read the diabetes policy” – Obstetrician 6, Policy maker 1*
(ii) Lack of communication between tertiary and primary care levels of care	*“The gap in the communication between a delivery unit and where the patient has to go to is one of our main concerns. So, this was meant to be the communication between the Delivery Unit and the primary healthcare facility where the mum and the baby are followed up” – Health services manager, Policy maker 2*
	*“They get a discharge summary in which we advise them to go to their local clinic in 6 weeks’ time to have their sugars checked but we don’t have any way of checking that it’s happened” – Professor, Obstetrician 2*
(iii) Inconsistencies in completion of referral letter	*“What we’ve discovered was that A it [the referral letter] doesn’t get completed very well and B for one or other reason, it doesn’t reach the primary healthcare clinics” – Health services manager, Policy maker 2*
(iv) Fragmentation of care	*“The key obstacle is that there’s such a divide between maternal and child care in the clinic setting, and the sisters are so habituated to that. They are either working on the maternal side, or they are working in the baby side” - Health Services manager, Policy maker 2*
	*“So certainly not all MOUs are going to be accessible to the patient, the community health centres might be the best place to do it [postpartum OGTT], but you’ve got to find out, can they cope?” – Professor, Obstetrician 4*
	*“To my knowledge, the Community Health Centre’s are not set up for it [postpartum OGTT]. The patient has to arrive early in the morning fasting. She’s then supposed to have a fasting blood sugar, and then 2 h afterwards, she’s supposed to have the 2 h blood sugar” Professor, Obstetrician 3*
(v) A foetal – centred approach to antenatal care	*“So the understanding that this is more of a lifestyle thing for the long-term future maybe isn’t there enough. It’s been very much geared around the pregnancy. I would say, our focus is the pregnancy, keep the sugar down, try and have a healthy baby and a mother that’s not injured during the birth. And we don’t think too much to the afterwards” – Professor, Obstetrician 2*
	*“I think it’s a good idea because this is a particular at risk population, who get good care during pregnancy and child birth and then often just disappear from the system” -Professor, Public health specialist 1*
Patient – related barriers	
(i) Perception of future risk of developing T2DM	*“Because they don’t feel ill so they tend not go to the clinic or the doctor when they don’t have an issue. So I don’t know what the barriers are but the clinics are available it just that they don’t go” – Obstetrician 6, Policy maker 1*
(ii) Non-attendance for postpartum OGTT	*“They are not lost to the system, they lose themselves from the system I would say. Each person has a responsibility to her own health. If you get all the information and you get the appointment, then the onus in on you” – Obstetrician 3* *“We do attempt to get them back for OGTTs, a small number do come back, but not all” – Professor, Obstetrician 1*
(iii) Resistance to long term dietary change	*“So there is quite a lot of resistance to dietary intervention and that’s probably the reason they don’t go back because they know somebody will just talk about their diet again” – Obstetrician 6, Policy maker 1*
	*“I can tell you, it happens here in hospital already, once that baby is born you’ll find the bottles of cool drinks, and then you tell her, you are promoting yourself to Insulin” - Diabetes Nurse*
(iv) Lack of time and cost of transport for postpartum follow-up visits	*“It might also be pie in the sky [expecting women to attend postpartum follow-up visits], because once you have a small one (a baby) at home, it’s very difficult to give up your time” – Professor, Obstetrician 4*
	*“It’s quite expensive when you think of what taxi fares they probably have to pay and they are all the ones that are the most at risk. The ones that haven’t got money for the transport to get back, and we invariably lose the most at risk 20, 25% of the people” – Professor, Public health specialist 2*
(v) Lack of agency to make lifestyle changes	*“If you’re an incredibly poor woman in a township with few choices, with a patriarchal man who takes control of your life and you have no choices, what’s your incentive to eat healthy and exercise? Really, it’s hard. So you must go home now, and don’t put sugar, don’t put salt, cut the gravy, no potatoes or whatever. They can’t do that: their husbands will have a hernia!” – Professor Public health specialist 2*
Theme 3: Views on integrated postnatal care for GDM women	
Concept of ‘One-Stop-Shop’	*“I think that integration in general is a really good idea. It is a no-brainer that we’ve missed for the past 100 years!” - General practitioner, Medical Anthropology Researcher*
	*“It’s such a good example of something of the ongoing care that’s needed and maybe it could even be applied to other areas like blood pressure or heart or whatever. It’s a really good concept” – Professor, Obstetrician 2*
	*“There isn’t a 6-week visit for the mum at the moment; it’s just for the baby. So we are trying to integrate that maternal and child health visit” – Obstetrician 6, Policy maker 1*
	*“I think today the emphasis is on holism, and a holistic approach to everything, and not just to concentrate on a single item which really upsets you”- Professor, Obstetrician 4*
Potential of leveraging an existing health service (i.e.; WBC)	*“It makes a lot of sense. It’s very nice that it’s integrated into something that exists and is standard practice” - Professor, Obstetrician 1*
	*“Excellent idea, because she will go for her baby...” – Obstetrician 3*
	*“Currently their focus postpartum is mainly on the baby. They do a developmental screening, immunise the baby, weigh it and check on nutrition; how’s the baby feeding and things like that. They tend to forget the mom, and that is what we specifically want to do with this postnatal policy” – Health Service manager, Policy maker 2*
	*“In my experience, the mother would rather take the baby for the 6-week visit than to go herself for anything if she is feeling well” – Professor, Obstetrician 2*
	*“Now, whether that can be done, I don’t know, to emphasise just the baby, and to then to say, well, you know, you’re a Diabetic, we’ll do an oral GTT at the same time, I’m not quite sure whether that’s the right approach. I think the idea is at least a step in the right direction, but whether she will come fasting is another question” - Professor, Obstetrician 4*
	*“I think it’s a good idea, if the mother didn’t have the baby, she wouldn’t go, but for the baby’s sake, she will go” – Diabetes nurse*
Theme 4: Feasibility of integrated postnatal care for GDM women in the WBC	
Resource constraints given the current clinic structure in the WBC	*“One of the core issues is that you’ve got a resource constrained situation, community health workers, nurses and even doctors are full to the brim. I mean their job is 120%, so anything else you give them, is a problem” – Professor, Public Health specialist 2*
	*“The OGTT is a 2 h test that involves administering glucose and that involves taking blood at those intervals. Quite simply, the primary care clinics are not going to cope with that. There are challenges in terms of staffing and costing and deficiencies need to be sorted out” – Obstetrician 5*
	*“I think one’s going to have to be careful with this integrated visit not to give too many tasks” – Professor, Obstetrician 2*
	*“It’s not that people are not aware that OGTTs need to be done, it is because the environment will be challenging for people to be doing OGTTs. That’s associated with human as well as financial resources” – Obstetrician 5*
Role of nurses	*“Ideally, to deliver the intervention, that person should be trained to do all of those things, so that it’s a kind of one-stop shop. I don’t know if it would be better to have an additional person [dietician]to do that nutrition counselling element who’s got some dietetics training but I don’t think we can have a dietician doing that, because we don’t have enough Dieticians to go around.”– General practitioner, Medical Anthropology Researcher*
	*“The nursing staff do the OGTT in any case. So the nursing staff at the Well Baby Clinic should be able to it” – Obstetrician 3*
	*“All nursing staff in South Africa, have been through General Nursing where they are exposed to all those things. So they are able to do it.” – Diabetes Nurse*
	*“I think a primary care nurse should be able to do it.” - Professor, Obstetrician 2*

**Table 2:** Key informant views on policies and clinical practice guidelines for the management of GDM

Available guidelines	Key Informant comments
A: International guidelines	
➢ National Institute for Health and Clinical Excellence (2015) [33]. Diabetes in Pregnancy: Management of Diabetes and its Complications from Preconception to the Postnatal Period. NICE Guideline NG3.	*“We follow international guidelines, I suppose more the NICE Guidelines from the UK but we adapt them for local context. The therapies we use are used internationally and then we try and adapt everything. You can’t use a London diet for our patients from Soweto” – Professor, Obstetrician 1*
➢ World Health Organization. (2013) [37]. Diagnostic Criteria and Classification of Hyperglycaemia First Detected in Pregnancy*.* Geneva, World Health Org. (WHO/NMH/MND/13.2).	*“From the beginning of 2014 we’ve been using the WHO criteria. We used to use the 100 g test, which was the United States test, but we changed in January last year, to 75 g” - Professor, Obstetrician 1* *“The new definition has made it easier. It said any altered carbohydrate metabolism, which included Impaired Glucose Tolerance; and then we used the WHO criteria from 1990” - Professor, Obstetrician 4* *“Everybody knows what to do with GDM - we all follow international standards and we all follow international protocol and recommendations within the resource constraints” – Obstetrician 5*
B. National guidelines	
➢ Guidelines for Maternity Care in South Africa. A manual for clinics, community health centres and district hospitals. 4th Ed. Pretoria: NDoH 2015: 172.	*“There would be something on diabetes and there is a national maternity care guideline” - Professor, Obstetrician 2*
C. Provincial guidelines	
Western Cape Department of Health policy guidelines	
➢ Diabetes in Pregnancy, Provincial guideline of the Western Cape, for the management of diabetes and its complications from pre-conception to the postnatal period (2010).	*“We have very little to do with implementation of services, but at our level, we do policies; we’ve got to write policies. We assist with guidelines and protocols, and what we do, is we monitor and evaluate the implantation of these guidelines and policies” – Health service manager, Policy maker 2* *“We do have a provincial policy - Circular 124 of 2010 which is due for review” – Health services manager, policy maker 2* *“Yes, there is a Western Cape province policy for diabetes in pregnancy which we put together. It’s about 5 years old so I think it needs revision but there haven’t been major changes so it will be minor revisions” – Obstetrician 6, Policy maker 1* *“So this would then be management of Diabetes during pregnancy. The policy speaks a little bit about what happens postnatally but not much,. We know that is where the gap is. We are currently busy doing a postnatal policy for the province” - Health services manager, Policy maker 2*
➢ Metro West Protocols for Basic Antenatal Care	*“So you will find in the City BANC Clinics, that they use our BANC protocols as well” – Health services manager, Policy maker 2*
➢ Midwife Obstetric Units (MOU) Protocols for clinical practice at level 1 maternity care facilities in the Metro West (PMNS)	*“There are the provincial guidelines and the protocol for each of the facilities is based on the overarching guideline” – Health services manager, Policy maker 2*
D. Hospital – clinical practice guidelines	
(i) Selective screening criteria for Gestational Diabetes (ii) Postpartum management of diabetic patients (iii) Information sheet and Meal plan	*“..there is a guideline for the tertiary hospital and that’s the one we follow in terms of management” - Professor, Obstetrician 2* *“…we just go with the guidelines that were produced by our department by our Professor, which I think he bases on some international opinion” – Obstetrician 5*
E. Evidence from empirical studies	
➢ ACHOIS study (Crowther et al.; 2005) [38]	*“We follow international guidelines on the management of Gestational Diabetes. It’s been proven in many studies, the HAPO and the ACHOIS studies, that you have to actually follow up people with just gestational or milder form of Gestational Diabetes as well, as this has an impact on the outcome of the baby” – Obstetrician 3*
➢ HAPO study (HAPO Study Cooperative Research Group, 2002) [39]	*“We are influenced quite a lot by recent research – such as the ACHOIS study, which was looking at GDM. For quite a long time, people didn’t think that impaired glucose tolerance was going to have a negative impact on the pregnancy” – Professor, Obstetrician 2*

### II. Perceived facilitators and barriers to lifestyle change

The majority of key informants felt that the mother’s concern for the health of the unborn baby was the single strongest facilitator for adherence to lifestyle modification during pregnancy. In particular, clinicians reported that in their experience, women with GDM were motivated to commit to lifestyle modification in order to deliver a healthy baby without complications, whilst others felt that fear of hospitalization for observation due to uncontrolled blood glucose also contributed to adherence to lifestyle modification.

Despite these facilitators, some clinicians, viewed their own counseling on dietary and lifestyle change during pregnancy as insufficient. While the three hospital study sites each have a dietician, whose role is to provide dietary education to GDM women, the informants bemoaned the high turnover and shortage of dieticians in the public health sector. As a result, doctors and nurses provide some dietary counseling during consultations, but high patient numbers and time constraints limit their ability to provide detailed educational information to each individual woman. In one of the hospitals, women have access to a dietician upon diagnosis of GDM. In another hospital, the dietician is only available once a week to provide group counseling to GDM women who are admitted to a hospital ward. Only one of the hospitals has diabetes nurse educators who are tasked with continuous counseling and patient education for this group of patients. Informants expressed a need for more updated and appropriate educational material on lifestyle modification, which they could use with GDM women.

Regarding physical activity as part of lifestyle intervention, many respondents commented that there was a greater focus on diet modification than the importance of increasing physical activity during pregnancy. Affordability of healthy food was recognised as a major challenge for women from low socioeconomic backgrounds, regardless of their desire to eat well. According to the public health specialists, unhealthy highly refined foodstuffs are often cheaper and more satisfying than the healthier options such as unprocessed starches and vegetables. Overall, despite these barriers, there was consensus among informants that women with GDM make a considerable effort towards lifestyle modification during pregnancy, although they suspected that these efforts were not usually sustained once the baby was born.

### III. Challenges with postpartum follow-up for GDM women

#### Health system barriers

Health service managers admitted that post-partum follow up for GDM women was problematic. They expressed concern over the fragmentation of health services (Fig. 2) and the absence of a standardised approach to postpartum follow-up care for GDM women. Postpartum care for the GDM mother and health care for the baby are currently available as two separate health services, which are not always located within the health facility. This inconvenience, they believed, posed a barrier to women attending for post-partum care. In addition, the OGTT is not routinely offered at the primary care level and no specific lifestyle advice is being delivered by these services to women with prior GDM. Our interviews revealed that only one of the hospitals in our study offers the 6 weeks postpartum OGTT in an effort to make it more convenient for women to access the OGTT. Even then, not all women attend, our key informants reported on average less than 50% attendance. Although it is standard practice to discharge the woman with a referral letter to a primary care clinic for an OGTT, this appears to be inconsistently done and there are no communication channels between the different levels of service that enable clinicians to verify whether a woman has had a post-partum OGTT.

#### Patient-related barriers

Because the majority of women who have had GDM are not on any diabetes medication when discharged, some participants believe they do not perceive themselves to be at risk and so fail to attend for postpartum follow-up care. They also noted that while women are highly motivated to eat more healthily during pregnancy, they were resistant to the idea of permanently altering their lifestyle. Two of the public health specialists were sceptical about achieving long term dietary change in this group of women in the context of patriarchal households where women have very little agency to make lifestyle changes for themselves, let alone the family.

One of the clinicians felt that where women were informed about the importance of the OGTT post-partum, they needed to take more responsibility for their own health and attend the follow-up visit. However, other key informants suggested that non-attendance could be due to lack of time, the cost of transport for postpartum visits and the fact that women may have returned to their rural home in another province. It is common for women residing in the Eastern Cape to migrate to the Western Cape province for the duration of their pregnancy and return home after delivery. Similar patterns of migration between urban and rural areas were reported by key informants in Gauteng. Key informants attributed this to the perception that women would have access to better quality of health services in the Western Cape. In light of these barriers to follow-up care and sustained lifestyle changes, we elicited key informant views on potential integrated postnatal care for women with prior GDM and their babies.

### IV. Views on feasibility of proposed intervention in the Well Baby Clinic

In general, all key informants supported the idea of integrated health services for all women after pregnancy. Policy makers reiterated that there is a need to bridge the divide between maternal and child health care services by means of a holistic approach to postnatal care. They were also in support of our proposed intervention which would potentially leverage the scheduled 6-week immunisation visit at the Well Baby clinics as an opportunity to conduct the OGTT and provide follow-up advice to the GDM mothers. At the time of the interviews, the provincial Western Cape Department of Health were in the process of finalising a postnatal care policy for all women. This new Western Cape Postnatal Care Policy [40] aims to provide integrated postnatal care (up to 6 weeks postpartum) for the mother and baby at the same visit, same site - in particular, the Well Baby Clinic, and by the same health provider.

Most participants felt that integrated care for mother and baby in the Well Baby Clinic would be feasible, if additional resources were made available. There was some concern about the cost implications (e.g.; human resources, medical supplies) for offering an OGTT in the Well Baby clinics. Some clinicians did not feel that the current set-up (i.e. space and clinic operations) in the Well Baby clinics could accommodate and attend to the health needs of both the mother and baby. Key informants also cautioned against overloading nursing staff and compromising the quality of care and affect morale. However, most were of the view that with resources, primary care nurses would be the ideal personnel to conduct the OGTT at the Well Baby clinics. Some suggested training community health workers or health promoters to provide counselling on lifestyle changes, whilst others insisted that nurses should trained to offer dietary counselling since they have frequent contact with patients. Due to the shortage of dieticians, none of the key informants felt that relying on a dietician would be a feasible option.

## Discussion

This study explored the policy guidelines and reported clinical practices relating to antenatal and post-natal care for women with GDM in South Africa, as well as health sector stakeholders’ perspectives on the patient and health systems barriers and opportunities for intervention. The health services in our study, adhere to international guidelines for screening, diagnosis and management of GDM– in particular, the WHO guidelines and an adaptation of the NICE guidelines. Further, antenatal care for women with GDM in the two provinces is intensive and in line with health policy, but postpartum care for GDM women appears to be poorly structured and misaligned with existing policy.

There is considerable international debate regarding the best approach to screening and diagnosis of GDM. The IADPSG criteria have been adopted by the WHO, which has different thresholds for diagnosis compared to the updated UK NICE guidelines [41]. As a result of the lack of consensus on screening and diagnostic criteria, there is no uniformity in screening practices [42, 43]. The WHO diagnostic criteria are generally accepted globally, including by many African countries such as Nigeria and Ethiopia [13, 44]. The WHO recommends that a GDM diagnosis be made at any time during pregnancy on the basis of a fasting plasma glucose value between 5.1-6.9 mmol and a 2-h post 75 g oral glucose load value of 8.5-11.0 mmol [37]. In low-resource settings, one of the challenges of this approach, aside from the costs associated with the actual OGTT, is that women may not always remember to come fasting for their ANC visit [45]. In most LMICs, including South Africa, where resources are limited, pregnant women are selectively screened for GDM on the basis of the presence of risk factors. Universal screening is not practiced in all high-income countries. For example, in the USA only some states follow this practice. [46, 47]. Further, a recent study found that universal screening for GDM in the UK was not cost-effective and of no added value in comparison to risk factor-based screening [41]. At this stage, there is no conclusive evidence as to which method is best practice, in fact, a recent review describes the GDM screening and diagnostic criteria as disorderly and chaotic [44].

According to our key informants, all primary care facilities providing antenatal care services should already have in place protocol guidelines for screening procedures and referral pathways for women at high risk for GDM. However, the extent to which this screening actually takes place and the sensitivity of these risk factors for identification of GDM is not known. It is possible that a proportion of likely GDM women either remain undiagnosed and therefore untreated, or are only diagnosed late in the pregnancy with possible adverse pregnancy outcomes. Sustainable Development Goal (SDG) 3 prioritises ‘strengthening the capacity of all countries, in particular developing countries, for early warning, risk reduction and management of national and global health risks’ [48]. For women of reproductive age, effective screening for GDM during pregnancy is necessary in order for them to receive appropriate antenatal care and ensure positive pregnancy outcomes. In addition, improving postpartum screening and follow-up care for women after GDM is critical to delaying or preventing progression to type 2 diabetes in this particular high-risk population [49, 50].

Our key informants reported that women with GDM are consistent in their attendance of antenatal care visits and make considerable effort towards lifestyle modification during pregnancy. However, a few key informants felt that women with GDM did not always demonstrate a clear understanding of what constituted a healthy diet. They expressed concern that they could not provide women with adequate counselling and educational information to empower them to make the necessary, long term lifestyle changes. According to an Australian qualitative study on GDM women’s experiences [51], in addition to partner support, support from health providers is also critical for women with GDM to make lifestyle changes. Key informants also mentioned that they detected a resistance to long-term dietary change among some GDM women and imagined that interventions in the post-partum period might be challenging - a view borne out in a systematic review of lifestyle interventions for GDM women [19]. Sustained diet modification is difficult for women with prior GDM women due to factors such as the unaffordability of healthy food, the absence of the pregnancy as a source of motivation and the lack of social support, especially from the family for dietary change once the pregnancy was over [10, 52–54]. Despite this, the initial diabetes prevention trials, which showed benefit in using intensive behaviour change interventions for people with pre-diabetes or impaired glucose tolerance [55], were also found to be just as effective among women with and without self-reported prior history of GDM [7, 49]. However, these challenges indicate the importance of conducting formative research to understand the context in which lifestyle modification is to be made. Our proposed lifestyle intervention for women with prior GDM would need to consider the particular barriers and facilitators for lifestyle change relevant to our target audience and offer recommendations for changes that are realistic and feasible [10].

Our study also highlights challenges with postpartum follow-up care for women with GDM which can be categorised as health systems barriers and patient-related barriers. The health systems barriers were similar to those cited by healthcare workers in other settings and included fragmentation of care and poor communication between health care workers delivering care at different levels [52, 56, 57]. Our respondents also discussed patient characteristics which appear to be common barriers to postpartum follow-up, such as lack of perceived future risk for developing type 2 diabetes and non-attendance for postpartum OGTT [10, 52]. The low perception of future risk for type 2 diabetes by women with GDM reported by our key informants may relate to the health system barriers which result in inadequate counselling and education during pregnancy. This is evident in clinicians not having the time during antenatal visits and the absence of a dietician to provide and reinforce lifestyle change counselling and education on GDM and the fact that physical activity is not adequately discussed with women despite its potential benefits [10]. Moreover, there is little emphasis during antenatal care on GDM being an opportunity to make long-term lifestyle changes which extend beyond the duration of the pregnancy. This may be attributed to health providers’ lack of appropriate training on lifestyle change counselling compounded by the shortage of dieticians in the public sector. Regardless, the unintended consequence of the foetal-centric approach versus a life-course approach to counselling is that women with GDM view the health of the unborn child as the main, if not only, incentive for lifestyle modification which does not extend beyond birth [58]. On the other hand, studies have found that where women perceive their risk of developing type 2 diabetes as immediate, they too may decide not to attend the 6-week postpartum screening out of fear of a diagnosis of diabetes and inadequate awareness that this risk can be modified [17, 56].

Non-attendance for postpartum follow up, is complex in that whilst it is a patient-related barrier, it is closely linked to (1) the quality of counselling and education women with GDM receive during pregnancy and (2) the implementation of health policy at primary care level once discharged. Several studies have found that even when women do attend for healthcare post-partum, many of them do not complete an OGTT [12, 52, 59, 60] either because it is not offered, or they do not request it. These findings emphasize the importance of training health providers and non-clinicians to offer high quality lifestyle change counselling and education to women with GDM, both to improve women’s understanding of the need for sustained lifestyle changes, as well as to facilitate long-term follow-up and [52, 54, 61].

Our findings confirmed that long term follow up in the postpartum period is problematic. Management of GDM in the postpartum period is unsatisfactory in comparison to during pregnancy where women receive intensive medical care from a specialised multidisciplinary medical team. This finding is not unique to South Africa; similar patterns have been reported elsewhere [8, 52, 62]. In this particular context, the absence of a standardized postnatal care approach for GDM women and the organization of primary health services may be creating an additional obstacle to GDM women accessing health services in the postpartum period – representing a missed opportunity for long term diabetes prevention care. Firstly, there is no system in place for the health providers at the delivery unit – i.e. the tertiary hospital to verify whether a woman with prior GDM has attended a follow-up visit and been offered an OGTT at her local community clinic. Discharge summaries which indicate the woman’s diagnosis and need for follow-up, seldom make it to the community clinics where women should attend for their postpartum screening for diabetes. In addition to improving communication between clinicians at the different levels of care, perhaps reminder systems for both patients and health providers could be utilized to improve rates postpartum follow-up and screening as suggested and demonstrated in previous studies [8, 52, 63, 64]. Results from a retrospective cohort study in the US suggest that GDM women are more likely to attend postpartum visits at a hospital-based clinic than at a hospital affiliated community clinic and this may be attributed to non-adherence to guidelines for postpartum follow-up care by health care providers at the community clinics [62].

Secondly, our respondents confirmed that maternal and child health services are currently two distinct services which the mother and baby have to navigate to access care. This set-up may be inconvenient and therefore contributing to women not seeking post-partum follow-up care for themselves after a GDM pregnancy yet still attending child health services for their baby. Compartmentalisation of care in the postpartum period is a known barrier to postpartum follow-up for GDM women [52]. It was a positive finding in our study that the policy makers and health service managers are cognisant of the inconsistencies between provision of antenatal and postpartum care for this group of women and are beginning address this through gradual policy change towards integrated postnatal care.

It is imperative that health systems improve their responsiveness and capacity to prevent and manage chronic diseases affecting reproductive-aged women. Fortunately, there is support among both decision makers and clinicians in SA for the idea of integrating a post-GDM type 2 diabetes prevention intervention into our public health system. Our key informants viewed the concept of integrated care for the mother and baby as an opportunity to improve postpartum care for women after GDM. Although they supported the proposed intervention in principle, a few expressed concerns regarding the feasibility of integrated health services given the resource constraints in our setting. However, given the high burden of type 2 diabetes in LMICs [65, 66], such health system interventions to support women with a history of GDM in making positive lifestyle changes that are sustainable in the long term are needed if high rates of progression to type 2 diabetes are to be avoided [66].

### Limitations

Due to the qualitative study design, generalisability of the findings may be limited. Our study results reflect the views of health stakeholders in the context of urban public health sector hospitals which may not be applicable to rural hospitals. However, the document review provided some triangulation of the interview findings in relation to policy guidelines for GDM management in SA. Further research on the perspectives of women with a history of GDM is necessary to ensure that the proposed intervention will be feasible and acceptable to its target population.

## Conclusions

The intensive antenatal care for GDM women in the study settings ensures high rates of positive delivery outcomes. However, there is currently a significant gap between the high standard of antenatal care for GDM women and services for women with GDM in the postpartum period. This formative qualitative study (1) provides a general overview of the management of GDM in South Africa; (2) highlights some of the main facilitators and challenges with lifestyle modification during pregnancy and (3) identifies important barriers and opportunities for postpartum intervention for women with a history of GDM. Finally, this study provides some useful formative research findings for the development of a complex intervention trial on type 2 diabetes prevention in women with a history of GDM and will assist in determining the most effective means of delivering a feasible and sustainable intervention for this high-risk group in the South African setting.

## Additional file

Additional file 1: Discussion guide for key informant interviews. Description of data: The discussion guide was used to direct discussions with key informants using probes. (PDF 62 kb)

## Abbreviations

BANC: Basic Antenatal Care
CHC: Community Health Centre
GDM: Gestational diabetes mellitus
IGT: Impaired Glucose Tolerance
MOU: Midwife Obstetric Unit
OGTT: Oral Glucose Tolerance Test
SA: South Africa
