# Supplementary material for: Policies and clinical practices relating to the management of gestational diabetes mellitus in the public health sector, South Africa – a qualitative study
Source: BMC Health Serv Res. 2018 May 10;18:349. doi: 10.1186/s12913-018-3175-x (PMC5946476; doi:10.1186/s12913-018-3175-x)
Supplement: Supplementary file 1 — Discussion guide for key informant interviews. Description of data: The discussion guide was used to direct discussions with key informants using probes. (PDF 62 kb) [file 12913_2018_3175_MOESM1_ESM.pdf]

An integrated health system intervention aimed at reducing type 2 diabetes risk in women after gestational diabetes in South Africa (IINDIAGO Study)

Key Informants Discussion Guide

<u>SECTION A: Standard of care for GDM women</u>

Policy & Clinical guidelines

1) What are the policies and clinical guidelines in place for the management of women with GDM and diabetes at this hospital?
   - When and how were they developed?
   - Who are the key implementers?
   - What is the oversight (Monitoring and Evaluation)?
   - How effective do you think they are?
   - How often do the women come to the hospital?
   - Who sees them?
   - What is the treatment regimen?

2) What are the policies and clinical guidelines relating to the care of the mother and baby after a GDM pregnancy?

3) What are the current in-hospital post-delivery guidelines for women with GDM and diabetes at this hospital?

4) To what extent are these policies and guidelines followed in clinical practice?

5) What are the problems (if any) in implementing these policies and guidelines?

6) What is currently happening at a policy level and within the health services regarding infant nutrition and vaccinations? How do you think this project can effectively align with these initiatives?

<u>SECTION B: Current practice</u>

7) Can you explain in detail the current practices relating to the care of women with GDM during pregnancy?
   - What lifestyle intervention do women receive during pregnancy?

   - What does it consist of? (Medication, educational materials etc.)
   - Who is involved in delivering the intervention?

8) What are the current practices/procedures relating to the care of women after a GDM pregnancy?

9) What is the current discharge plan for these women postpartum?

   - Is there a specific protocol in place?
   - Is there a referral system?
   - Where do women actually attend for postpartum follow-up for their diabetes or GDM?
   - Are women discharged on medication?
   - Is there a link between GDM management and labour/delivery procedures?

Prioritization of GDM in overall care

10) Do you regard postpartum follow-up of women with GDM as an issue needing to be addressed?

11) Is there anyone else who you think we would need to speak to as part of the formative phase of this study?

12) What do you think should be done for women with previous GDM during postpartum care?

13) What are your thoughts and feelings on integration of mother and baby care after a GDM pregnancy?

SECTION C: Views on proposed intervention

14) You have been given the draft protocol to read, do you have any critical comments/concerns you would like to raise about the proposed study? This could be regarding the conceptualization and feasibility of the research aspects and/or the proposed intervention? Do you have any additions to the protocol?

15) How feasible is a maternal health intervention in the Well Baby Clinic? (OGTT + Lifestyle intervention) Is there enough space/personnel/time/equipment/material to implement it?

16) Do you know if there are any other initiatives like this (integrated mother-baby care) in SA or elsewhere? Do you know of any follow-up interventions for women with previous GDM?
